# Development and Validation of Novel Deep-Learning Models Using Multiple Data Types for Lung Cancer Survival

**DOI:** 10.3390/cancers14225562

**Published:** 2022-11-12

**Authors:** Jason C. Hsu, Phung-Anh Nguyen, Phan Thanh Phuc, Tsai-Chih Lo, Min-Huei Hsu, Min-Shu Hsieh, Nguyen Quoc Khanh Le, Chi-Tsun Cheng, Tzu-Hao Chang, Cheng-Yu Chen

**Affiliations:** 1 Clinical Data Center, Office of Data Science, Taipei Medical University, Taipei 110, Taiwan; 2 Clinical Big Data Research Center, Taipei Medical University Hospital, Taipei Medical University, Taipei 110, Taiwan; 3 Research Center of Health Care Industry Data Science, College of Management, Taipei Medical University, Taipei 110, Taiwan; 4 International Ph.D. Program in Biotech and Healthcare Management, College of Management, Taipei Medical University, Taipei 110, Taiwan; 5 Graduate Institute of Biomedical Informatics, College of Medical Science and Technology, Taipei Medical University, 250 Wu-Hsing Str., Xinyi Dist., Taipei 110, Taiwan; 6 Office of Data Science, Taipei Medical University, Taipei 110, Taiwan; 7 Graduate Institute of Data Science, College of Management, Taipei Medical University, Taipei 110, Taiwan; 8 Department of Pathology, National Taiwan University Hospital, Taipei 100, Taiwan; 9 Graduate Institute of Pathology, College of Medicine, National Taiwan University, Taipei 100, Taiwan; 10 Professional Master Program in Artificial Intelligence in Medicine, College of Medicine, Taipei Medical University, Taipei 110, Taiwan; 11 Research Center for Artificial Intelligence in Medicine, Taipei Medical University, Taipei 110, Taiwan; 12 Department of Radiology, College of Medicine, Taipei Medical University, 250 Wu-Hsing Str., Xinyi Dist., Taipei 110, Taiwan

**Keywords:** lung cancer, survival, prediction models, real-world data, artificial intelligence, machine learning

## Abstract

**Simple Summary:**

Previous survival-prediction studies have had several limitations, such as a lack of comprehensive clinical data types, testing only in limited machine-learning algorithms, or a lack of a sufficient external testing set. This lung-cancer-survival-prediction model is based on multiple data types, multiple novel machine-learning algorithms, and external testing. This predicted model demonstrated a higher performance (ANN, AUC, 0.89; accuracy, 0.82; precision, 0.91) than previous similar studies.

**Abstract:**

A well-established lung-cancer-survival-prediction model that relies on multiple data types, multiple novel machine-learning algorithms, and external testing is absent in the literature. This study aims to address this gap and determine the critical factors of lung cancer survival. We selected non-small-cell lung cancer patients from a retrospective dataset of the Taipei Medical University Clinical Research Database and Taiwan Cancer Registry between January 2008 and December 2018. All patients were monitored from the index date of cancer diagnosis until the event of death. Variables, including demographics, comorbidities, medications, laboratories, and patient gene tests, were used. Nine machine-learning algorithms with various modes were used. The performance of the algorithms was measured by the area under the receiver operating characteristic curve (AUC). In total, 3714 patients were included. The best performance of the artificial neural network (ANN) model was achieved when integrating all variables with the AUC, accuracy, precision, recall, and F1-score of 0.89, 0.82, 0.91, 0.75, and 0.65, respectively. The most important features were cancer stage, cancer size, age of diagnosis, smoking, drinking status, EGFR gene, and body mass index. Overall, the ANN model improved predictive performance when integrating different data types.

## 1. Introduction

Lung cancer is the leading cause of cancer deaths worldwide [1]. Globally, there were around 2.21 million new cases of lung cancer and 1.80 million fatalities in 2020 [2]. One study reported that lung cancer incidence and mortality rates were 22.2 and 18.0 per 100,000 people in 2020, respectively [3,4]. Lung cancer can be divided clinically into two types based on histological features: non-small-cell lung cancer (NSCLC) and small-cell lung cancer (SCLC). NSCLC is the most common among them, accounting for 80–90% of lung cancers [5]. Cell deterioration and metastasis are slower in NSCLC than in SCLC. Around 70% of patients are diagnosed at an advanced stage, making surgical resection and complete treatment challenging [6,7].

Artificial intelligence (AI) has been increasingly used in medical research and clinical practice [8,9]. The accurate prediction of disease prognosis and the outcome of drug treatment, which may serve as a reference for treatment decision-making and drug selection, has become an essential topic in the clinical medicine [9,10]. Developing disease-risk and prognosis-prediction models using machine-learning or deep-learning algorithms with big data is a major area of AI-based academic research in the medical field [10,11]. Studies have used machine-learning and/or deep-learning algorithms to develop lung cancer risk and prognosis-prediction models [12,13,14,15]. Among them, Lai et al. [16] used 15 biomarkers with clinical data (including gene expression) from 614 patients to develop a deep neural network to predict the five-year overall survival of NSCLC patients.

This study aimed to develop survival-prediction models for lung cancer patients using a large number of samples, different data types, various machine-learning algorithms, and external testing. In addition to the basic clinical data (including demographic information, disease condition, comorbidity, and current medication), we examined the role of laboratory and genomic test results, which are generally not easy to obtain in predicting lung cancer survival. Moreover, we also explored the important predictors for developing prediction models.

## 2. Methods

### 2.1. Study Design and Data Source

We conducted a retrospective study in which we obtained data from the Taiwan Cancer Registry (TCR) database and the Taipei Medical University Clinical Research Database (TMUCRD). The TCR database was established in 1979 and is managed by Taiwan’s Health Promotion Administration, Ministry of Health and Welfare. It covers 98% of Taiwanese cancer patients and includes diagnosis and other related information. The TMUCRD retrieved data from various electronic medical records (EHR) of three hospitals, Taipei Medical University Hospital (TMUH), Wan-Fang Hospital (WFH), and Shuang-Ho Hospital (SHH). The database contains the electronic medical record data of 3.8 million people from 1998 to 2020, including structured data (e.g., basic information of patients, medical information, test reports, diagnosis results, treatment process, surgery, and medication history) and unstructured data (e.g., progress notes, pathology reports, and medical imaging reports) [17]. This study has been approved by the Joint Institute Review Board of Taipei Medical University (TMU-JIRB), Taipei, Taiwan (approval number N202101080). All the data were anonymous before conducting analysis.

### 2.2. Cohort Selection

This study selected patients with lung cancer (ICD-O-3 code: C33, C34) from 2008 to 2018 in the TCR database. Exclusion criteria included individuals under 20 years old, SCLC patients, and patients who did not have any medical history in the three hospitals (TMUH, WFH, SHH). Thus, a total of 3714 patients were included in this study, including 960 patients from TMUH, 1320 from WFH, and 1434 from SHH (Figure S1 in the Supplementary Materials).

### 2.3. Outcome Measurement

We ascertained the study outcomes using TMUCRD EHR and vital status data from the Taiwan Death Registry (TDR) [18]. We used the diagnosis date of NSCLC as the index date, and the outcome of this study was death within two years following diagnosis. Data were censored at the date of death or loss to follow-up, insurance termination, or the study’s end on 31 December 2018.

### 2.4. Feature Selection

Based on a literature review and consultation with clinicians, we selected features that may lead to the mortality of NSCLC patients to build prediction models. These features consisted of:

1. Demographic information: age, gender, body mass index (BMI), smoking, drinking;
2. Cancer conditions: tumor size and cancer stage;
3. Comorbidities: cardiovascular problems (i.e., myocardial infarction (MI), congestive heart failure (CHF), peripheral vascular disease (PVD), and cardiovascular disease (CVD)), dementia, chronic obstructive pulmonary disease (COPD), rheumatic disease, peptic ulcer disease (PUD), renal disease, liver disease, diabetes, anemia, depression, hyperlipidemia, hypertension, Parkinson’s disease, and Charlson Comorbidity Index (CCI) score. These conditions were considered if they were diagnosed in at least two outpatient claims or one hospitalization over a year before the cancer diagnosis date.
4. Medications: alimentary tract and metabolism, blood and blood-forming organs, cardiovascular system, genitourinary system and hormones, musculoskeletal system, nervous system, and respiratory system. We measured patients who had used medications by receiving them for more than a month (i.e., 30 days) during a year (i.e., 360 days) before the index date.
5. Laboratory tests: basophil, blood urea nitrogen (BUN), calcium, cholesterol, chloride, creatinine, eosinophil, ferritin, glucose AC, HbA1c, HCT, HGB, potassium, lymphocyte, MCH, MCHC, MCV, monocyte, sodium, neutrophil, platelet (PLT), RBC, triglyceride, and WBC. We only selected laboratory tests with a missing rate of less than 70% values a year before or a month after the index date.
6. Genomic tests: ALK, EGFR, KRAS, PDL1, and ROS1. We collected genomic tests if patients had ever taken one a month after the cancer diagnosis date.

### 2.5. Development of the Algorithms

This study established prediction models based on four modes and different algorithms:

- The primary mode (e.g., Mode 1) included demographic information, cancer conditions, comorbidities, and medications.
- The second mode (Mode 2) included the data from Mode 1 and the laboratory tests.
- The third mode (Mode 3) included the data from Mode 1 and genomic tests.
- The fourth mode (Mode 4) considered all the above features.

This study aims to predict the survival of lung cancer patients; therefore, the problem can be formulated as a classification model as it could occur in the same patients. We used possible machine-learning techniques such as logistic regression (LR), linear discriminant analysis (LDA), light gradient-boosting machine (LGBM), gradient-boosting machine (GBM), extreme gradient boosting (XGBoost), random forest (RF), AdaBoost, support vector machine (SVC), and artificial neural network (ANN). These methods are briefly introduced below.

Logistic Regression (LR): This is a discrete choice model that models the relationship between a response and multiple explanatory variables and is based on the concept of probability [19]. It is widely used and more practical in fields such as biostatistics, clinical medicine, and quantitative psychology. Its Equation (1) is:

(1) $$y=\frac{e^{\left(b_{0}+b_{1}X\right)}}{1+e^{\left(b_{0}+b_{1}X\right)}\;}$$

where *x* is the input value, *y* is the predicted output, *b*_0_ is the bias or intercept term, and *b*_1_ is the coefficient for input (*x*). In this study, we used the LR function with the parameter C (inverse of regularization strength) of 0.0001 to reduce the model’s overfitting.

Linear Discriminant Analysis (LDA): This is generally used to classify patterns between two classes; however, it can be extended to multiple patterns. LDA assumes that all classes are linearly separable, and according to the multiple linear discrimination functions representing several hyperplanes in the feature space are created to distinguish the classes [20]. In this study, we set the parameters’ *shrinkage* to ‘0’ and the *solver* to ‘lsqr’ to improve estimation and classification accuracy.

Light Gradient-Boosting Machine (LGBM): This is a gradient-boosting framework that uses tree-based learning algorithms. It is designed to be distributed and efficient with the following advantages: faster training speed and higher efficiency; lower memory usage; better accuracy; support of parallel, distributed, and GPU learning; and capability to handle large-scale data [21]. The model’s *class_weight* parameter was set as ‘balanced’, which uses the output’s value to automatically adjust weights inversely proportional to class frequencies in the input data. The *learning_rate*, l1 regularization—*reg_alpha*, and l2 regularization—*reg_lambda* parameters were set as 0.05, 0.1, and 0.1, respectively.

Gradient-Boosting Machine (GBM): Gradient-boosting regression trees produce competitive, highly robust, and interpretable procedures for regression and classification. The ability of TreeBoost procedures to give a quick indication of potential predictability, coupled with their extreme robustness, makes them a useful preprocessing tool that can be applied to imperfect data [22]. The default parameters were used in this model.

Extreme Gradient Boosting (XGBoost): XGBoost, an efficient and scalable implementation of the gradient-boosting framework, is a machine-learning system for tree boosting. The scalability of XGBoost is attributed to several critical systems and algorithmic optimizations. These innovations include a novel tree-learning algorithm for handling sparse data; a theoretically justified weighted quantile sketch procedure allows the handling of instance weights in approximate tree learning [23]. The default parameters were used in this model.

Random Forest (RF): RF is an ensemble-learning method that operates by constructing many small scales of classification modules (most often decision trees) at the training time. The model outputs the class that combines the result of the individual modules based on some voting algorithms [24]. In this study, we set the parameters as follows: *n_estimators* (the number of trees) of 500, *max_depth* of 10, *min_samples_split* of 400, and *class_weight* of 0.5 for each class.

AdaBoost: The AdaBoost algorithm is an iterative procedure that combines several weak classifiers to approximate the Bayes classifier C∗(*x*). AdaBoost builds a classifier, e.g., a classification tree that produces class labels, starting with the unweighted training sample. If a training data point is misclassified, the weight of that data point is increased (boosted). A second classifier is built using the new weights, which are no longer equal. Again, misclassified training data have their weights boosted, and the procedure is repeated [25]. The number of estimators (*n_estimators*) used was 100.

Support Vector Machine (SVC): This is a machine-learning algorithm that can be applied to linear and nonlinear data. SVC transforms the original data to a higher dimension, from which it can use the super vectors in the training data set to find the hyperplane for categorizing the data. An SVC mainly identifies the hyperplane with the most significant margin, e.g., the maximum marginal hyperplane, to achieve higher accuracy [26]. The SVC can be represented by the following Equation (2):

(2) $$f\left(x\right)=\overset{N}{\underset{i=1}{\sum }}\left(\alpha_{i}^{*}-\alpha_{i}\right)K\left(x,x_{i}\right)+Β$$

where $K\left(x,x_{i}\right)$ is the kernel function, $\alpha_{i},\alpha_{i}^{*}\geq 0$ are the Lagrange multipliers, and Β is a bias term. In this study, we used a *linear* kernel for computations.

Artificial Neural Network (ANN): This is a learning algorithm vaguely inspired by biological neural networks. Computations are structured in terms of an interconnected group of artificial neurons, and these neutrons process information using a connectionist approach to computation. They are usually used to model complex relationships between inputs and outputs, find patterns in data, or capture the statistical structure [27]. The number of hidden layers with the number of neurons in each layer was set at 3 and 16, respectively. Additionally, for each layer, the *l2 regularization* of 0.01 and the ‘relu’ *activation* were used in the study. We set the ‘softmax’ activation for the output layer. We also used the ‘Adam’ *optimizer*, a highly performant stochastic gradient descent algorithm, and ‘binary_crossentropy’ as the binary classification outcome for the *loss* function.

### 2.6. Evaluating the Algorithms

The training dataset contained the data of patients from TMUH and WFH. The stratified 5-fold cross-validation was applied in the training set to assess the different machine-learning models’ performance and general errors. In other words, patients in the training set were divided into five groups, each used repeatedly as the internal validation set. We recruited data from SHH and used it for the external testing dataset to generalize the model.

The performance of the algorithms was measured by the area under the receiver operating characteristic curve (AUC), accuracy, sensitivity (recall), specificity, positive predictive value (PPV, precision), negative predictive value (NPV), and F1-score. We defined the best model using the highest AUC by comparing various models based on the external testing set. Furthermore, we analyzed the feature’s contribution (i.e., the feature’s importance) of the best model using SHAP values (SHapley Additive exPlanations) [28].

All the data processing was performed using MSSQL server 2017 (Redmond, WA, USA), and the model training and testing were performed using Python version 3.8 (Wilmington, DE, USA) with scikit-learn version 1.1 (Paris, France) [29].

## 3. Results

### 3.1. Baseline Characteristics of Patients

We identified 3714 eligible lung cancer patients diagnosed for the first time and registered at the TCR. Among those patients, 2280 patients were included in the training dataset, whereas 1434 were in the testing dataset. Demographic characteristics, comorbidities, tumor size, tumor stage, genomic tests, medication uses, and laboratory tests are presented in Table 1. The mean (standard deviation, SD) ages and BMI of cohort patients were 68 (13.7) and 23.4 (4.33), respectively. Most of the patients were male (57.5%) with late-stage lung cancer (i.e., stage IV, 54.8%), and patients were less likely to smoke (26.7%) or drink (11%). The cohort of patients had comorbidities related to hypertension (19.8%), hyperlipidemia (13.9%), COPD (16.1%), and CVD problems (11.6%). The follow-up durations for the cohort patients were a mean (SD) of 2.25 (2.47) years and a median (interquartile range (IQR)) of 1.41 [0.46–3.04] years. Detailed information is shown in Table S1 in the Supplementary Materials.

### 3.2. The Performances of Different Prediction Models

**Figure 1:**
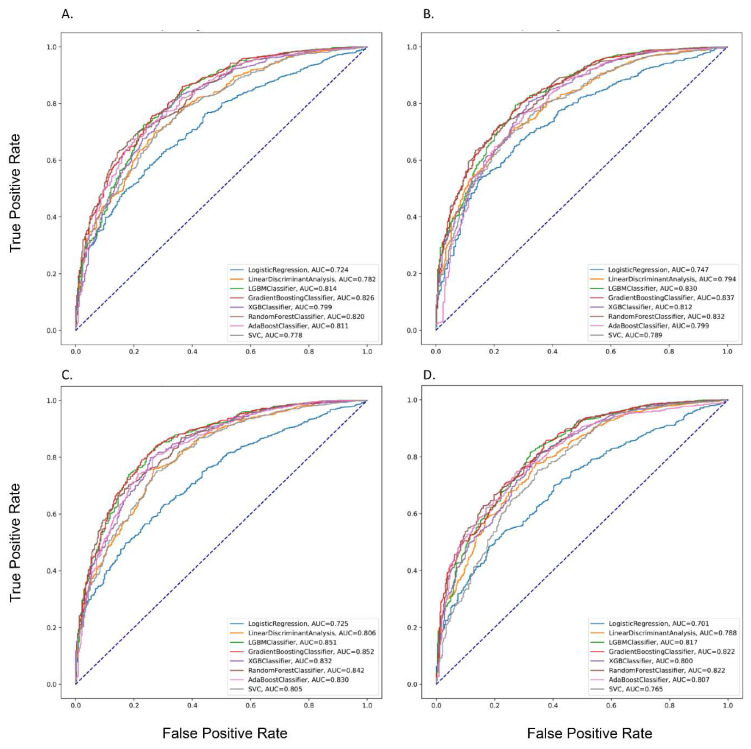
The Performance of the Prediction Models in the Testing dataset by different Modes. **Note**: (**A**), Mode 1; (**B**), Mode 2; (**C**), Mode 3; (**D**), Mode 4.

**Figure 2:**
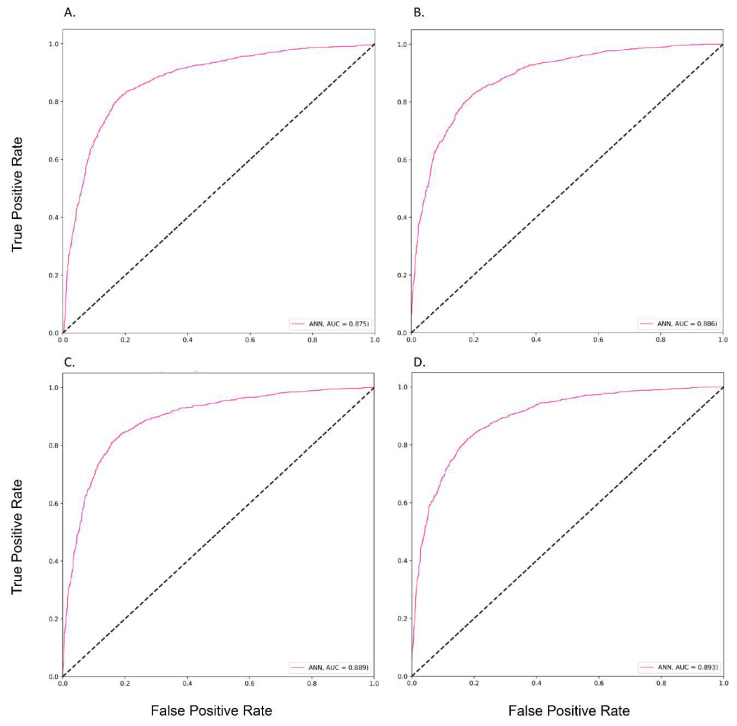
The Performance of the ANN Prediction Models in the Testing dataset by different Modes. **Note**: (**A**), Mode 1; (**B**), Mode 2; (**C**), Mode 3; (**D**), Mode 4.

The performances of different prediction models are shown in Table 2. In Mode 1, the highest AUC of 0.88 was observed for the ANN model (i.e., accuracy, 0.82; precision, 0.90; recall, 0.75; and F1-score, 0.64), followed by the GBM and RF models with an AUC of 0.83 and 0.82, respectively. In Mode 3, the best performance was found with an AUC of 0.89 for the ANN model (i.e., accuracy, 0.83; precision, 0.89; recall, 0.81; and F1-score, 0.64). The following AUCs were observed 0.85 for LGBM, GBM, and 0.84 for RF models. Moreover, when considering all features in Mode 4, we found that the best model was the ANN model with an AUC of 0.89 (i.e., accuracy, 0.82; precision, 0.91; recall, 0.75; and F1-score, 0.65). Figure 1 and Figure 2 show the ROC curves of different prediction models in four modes. Detailed information on the various models’ measurements (i.e., sensitivity, specificity, PPV, NPV, accuracy, and F1-score) is shown in Table S2 in the Supplementary Materials.

**Figure 3:**
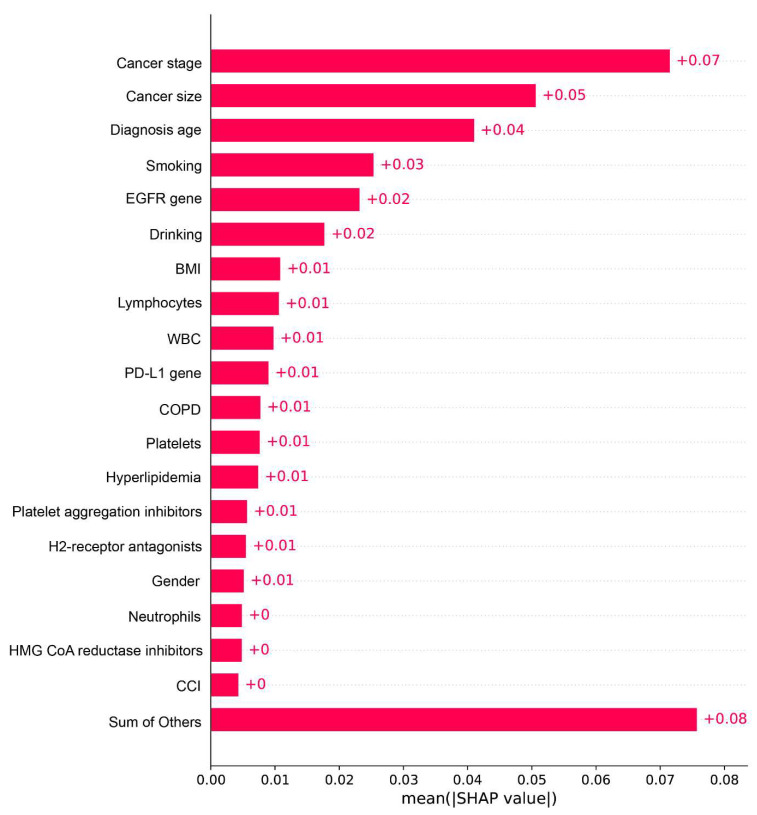
Feature Importance of the ANN Prediction Model in Mode 4. **Note**: BMI, Body mass index; EGFR, Epidermal growth factor receptor; WBC, White blood cell; PD-L1, Programmed death-ligand 1; COPD, Chronic obstructive pulmonary disease; CCI, Charlson comorbidity index.

Figure 3 shows the top 20 important features of the ANN model in Mode 4. The most important features were the cancer stage, size, age of diagnosis, smoking, and EGFR gene. In other words, patients with advanced cancer stage, large cancer size, older age, and smoking behavior had a higher risk of death within two years. The SHAP value presented the important features of the GBM model in Mode 4 and was consistent with the ANN model, such as cancer stage, age at diagnosis, cancer size, and smoking status (Figure S2 in the Supplementary Materials).

## 4. Discussion

In recent years, the prediction of cancer patients’ survival has attracted the medical community’s attention in various countries because it can facilitate medical decision making, strengthen the relationship between doctors and patients, and improve the quality of medical care. Rapid progress in the development of AI based on machine learning has led to more diversified applications of AI in the field of precision medicine. Based on previously published studies on machine-learning algorithms to build prediction models for the survival of lung cancer patients [12,14,15,16], this study further compared the performance of various novel machine-learning algorithms. In addition, we also analyzed the relationship between the diversity of features and the accuracy of prediction results and determined the most important features affecting lung cancer survival.

Studies using multiple data types and multiple novel machine-learning algorithms simultaneously are limited. In previous studies on lung cancer prediction, most of them used a single machine-learning (e.g., RF [30]) or deep-learning (e.g., NN [14,15,16]) algorithm or a few basic machine-learning algorithms (e.g., LR, SVM, decision tree, RF, GBM [12,31]) to develop prediction models. Our results showed that the ANN model had the highest AUC value (it was the most suitable tool for survival prediction). In contrast, the AUC value of the traditional LR algorithm exhibited the lowest performance (it had the lowest predictive ability). Lai Y.H. et al. [16] presented a deep neural network to predict the overall survival of NSCLC patients. They obtained a good predictive performance (AUC = 0.82, accuracy = 75.4%) by integrating microarray and clinical data. While only using basic clinical data (demographics, comorbidities, and medications), our predicted model demonstrated a higher performance (ANN, AUC, 0.88; accuracy, 0.82; precision, 0.90, recall, 0.75, and F1-score, 0.64). Furthermore, when combining other variables, such as laboratory and genomic tests, the AUC values of the predicted model were better (based on the external testing, the AUCs of the ANN model in Mode 1 and Mode 4 were 0.88 and 0.89, respectively; the AUCs of LGBM model in Mode 1 and Mode 4 were 0.81 and 0.86, respectively; the AUCs of the RF model in Mode 1 and Mode 4 were 0.82 and 0.85, respectively).

In this study, we explored the variables that might affect the predictive performance of the survival model. As expected, these variables were highly correlated to the mortality of lung cancer patients, such as advanced cancer stage, tumor size, age at diagnosis, and smoking and drinking status [32]. Our findings also showed that lymphocytes, platelets, and neutrophils tests were associated with the likelihood of lung cancer survival [33]. Thus, lymphocytes play an essential role in producing cytokines, inhibiting the proliferation of cancer cells, and provoking cytotoxic cell death [34]. In words, a decrease in lymphocyte count may predict worse survival in cancer patients. Neutrophils are recruited with cytokines released by the tumor microenvironment, enhancing carcinogenesis and cancer progression [35]. Platelets modulate the tumor microenvironment by releasing factors contributing to tumor growth, invasion, and angiogenesis [36]. Another study by Wang J. et al. [37] reported that lung cancer patients with a higher BMI have prolonged survival compared to those with a lower BMI. The same was true for our study’s results, which may be due to the poor nutrition and weight loss caused by respiratory diseases [38], such as COPD.

There are limitations to this study. First, although the study used data from various clinical settings (e.g., TMUH and WFH for establishing the prediction model and SHH for conducting an external test) located in the north of Taiwan, the results may not directly apply to lung cancer patients in other regions. Future studies may need to consider validating the model using data from other areas. Second, this study used retrospective data for development and validation. Further experiments with a prospective study design in clinical settings are needed. Third, to obtain a highly accurate prediction, we developed the machine-learning algorithms with binary outcomes (i.e., survival and death) rather than expected continuous outcomes (i.e., length of survival) for the NSCLC patients. Further studies should be conducted with larger sample sizes to deal with continuous outcomes for lung cancer survival.

## 5. Conclusions

In summary, to observe the expected survival of NSCLC patients during a two-year period, we designed an artificial neural network model with high AUC, precision, and recall. Moreover, integrating different data types (especially laboratory and genomic data) led to better predictive performance. Further research is necessary to determine the feasibility of applying the algorithm in the clinical setting and explore whether this tool could improve care and outcomes.

## Figures and Tables

**Table 1:** Basic Characteristics of the Study Cohort.

Features	Overall *n* = 3714	Training Set ^a^ *n* = 2280	Testing Set ^b^ *n* = 1434
**Male, N (%)**	2136 (57.5)	1258 (55.2)	878 (61.2)
**Age, Mean (SD), yrs.**	68.0 (13.7)	67.9 (13.8)	68.0 (13.4)
**BMI, Mean (SD), kg/m^2^**	23.4 (4.33)	23.4 (3.93)	23.4 (4.81)
**Smoking, N (%)**			
No	1170 (31.5)	710 (31.1)	460 (32.1)
Yes	993 (26.7)	523 (22.9)	470 (32.8)
Unknown	1551 (41.8)	1047 (45.9)	504 (35.1)
**Drinking, N (%)**			
No	1750 (47.1)	983 (43.1)	767 (53.5)
Yes	408 (11.0)	247 (10.8)	161 (11.2)
Unknown	1556 (41.9)	1050 (46.1)	506 (35.3)
**Tumor size, cm**			
Mean (SD)	4.23 (2.45)	4.11 (2.39)	4.46 (2.55)
Median [IQR]	3.8 [2.4–5.5]	3.6 [2.3–5.5]	4.0 [2.5–5.7]
**Cancer stage, N (%)**			
0	11 (0.3)	10 (0.4)	1 (0.1)
I	533 (14.4)	348 (15.3)	185 (12.9)
II	139 (3.7)	88 (3.9)	51 (3.6)
III	527 (14.1)	330 (14.5)	197 (13.7)
IV	2034 (54.8)	1207 (52.9)	827 (57.7)
Missing	470 (12.7)	297 (13.0)	173 (12.1)
**Genomic Test**			
**ALK, N (%)**			
Negative	681 (18.3)	457 (20.0)	224 (15.6)
Positive	39 (1.1)	21 (0.9)	18 (1.3)
Unknown	2994 (80.6)	1802 (79.0)	1192 (83.1)
**EGFR, N (%)**			
Negative	842 (22.7)	473 (20.7)	369 (25.7)
Positive	787 (21.2)	467 (20.5)	320 (22.3)
Unknown	2085 (56.1)	1340 (58.8)	745 (52.0)
**KRAS, N (%)**			
Negative	45 (1.2)	32 (1.4)	13 (0.9)
Positive	5 (0.1)	2 (0.1)	3 (0.2)
Unknown	3664 (98.7)	2246 (98.5)	1418 (98.9)
**PDL1, N (%)**			
Negative	269 (7.2)	149 (6.5)	120 (8.4)
Positive	66 (1.8)	42 (1.8)	24 (1.7)
Unknown	3379 (91.0)	2089 (91.6)	1290 (90.0)
**ROS1, N (%)**			
Negative	288 (7.8)	287 (12.6)	1 (0.1)
Positive	29 (0.8)	27 (1.2)	2 (0.1)
Unknown	3397 (91.4)	1966 (86.2)	1431 (99.8)
**Comorbidity, N (%)**			
CVD problems	432 (11.6)	296 (13.0)	136 (9.5)
Dementia	124 (3.3)	71 (3.1)	53 (3.7)
COPD	599 (16.1)	391 (17.1)	208 (14.5)
Rheumatic disease	28 (0.75)	16 (0.7)	12 (0.8)
PUD	365 (9.8)	246 (10.8)	119 (8.3)
Renal disease	128 (3.4)	92 (4.0)	31 (2.2)
Liver disease	211 (5.7)	147 (6.4)	64 (4.5)
DM	372 (10.0)	248 (10.9)	124 (8.6)
Anemia	107 (2.9)	76 (3.3)	31 (2.2)
Depression	245 (6.6)	175 (7.7)	70 (4.9)
Hyperlipidemia	516 (13.9)	385 (16.9)	131 (9.1)
Hypertension	736 (19.8)	503 (22.1)	233 (16.2)
Parkinson’s disease	50 (1.3)	29 (1.3)	21 (1.5)
**Charlson Comorbidity Index (CCI)**			
Mean (SD)	3.08 (2.07)	3.13 (2.19)	2.97 (1.86)
Median [IQR]	3.0 [2.0–4.0]	3.0 [2.0–4.0]	3.0 [2.0–4.0]
**Follow-up, yrs.**			
Mean (SD)	2.25 (2.47)	2.44 (2.61)	1.96 (2.19)
Median [IQR]	1.41 [0.46–3.04]	1.51 [0.53–3.36]	1.24 [0.38–2.64]
**Medications, N (%)**			
Alimentary tract and metabolism	591 (15.9)	394 (17.3)	197 (14.7)
Blood and blood-forming organs	446 (12.0)	293 (12.9)	153 (11.3)
Cardiovascular system	675 (18.2)	448 (19.6)	227 (16.9)
Genitourinary system and hormones	132 (3.6)	74 (3.2)	58 (4.3)
Musculoskeletal system	252 (6.8)	141 (6.2)	111 (8.3)
Nervous system	391 (10.5)	254 (11.1)	137 (10.2)
Respiratory system	319 (8.6)	226 (9.9)	93 (6.9)
**Laboratory Test, Mean (SD)**			
Basophil	0.50 (0.40)	0.53 (0.42)	0.48 (0.39)
BUN	19.4 (14.9)	18.8 (13.1)	20.5 (17.6)
Creatinine	1.05 (0.98)	1.02 (0.90)	1.10 (1.07)
Eosinophil	1.89 (2.31)	2.03 (2.59)	1.76 (1.97)
HCT	38.3 (5.69)	38.5 (5.61)	37.9 (5.80)
HGB	12.9 (1.97)	13.0 (1.91)	12.7 (2.05)
K	3.99 (0.56)	4.02 (0.53)	3.95 (0.60)
Lymphocyte	18.7 (9.98)	19.6 (9.55)	17.8 (10.3)
MCH	29.9 (3.02)	29.9 (3.03)	29.8 (3.00)
MCHC	33.6 (0.95)	33.7 (0.96)	33.6 (0.94)
MCV	88.6 (7.61)	88.5 (7.64)	88.7 (7.57)
Monocyte	7.45 (2.90)	7.42 (2.93)	7.48 (2.87)
Na	137 (4.46)	137 (4.39)	137 (4.53)
Neutrophil	71.3 (11.9)	70.2 (11.4)	72.3 (12.2)
PLT	263 (109)	258 (100)	269 (121)
RBC	4.35 (0.68)	4.38 (0.67)	4.29 (0.69)
WBC	9.72 (5.38)	9.16 (4.16)	10.6 (6.80)

**Note**: SD, Standard deviation; yrs., Years; IQR, Interquartile Range; BMI, Body mass index; COPD, Chronic obstructive pulmonary disease; PUD, Peptic ulcer disease; CVD, Cardiovascular; DM, Diabetes; BUN, Blood urea nitrogen; HCT, Hematocrit; HGB, Hemoglobin; K, Potassium; MCH, Mean corpuscular hemoglobin; MCHC, Mean corpuscular hemoglobin concentration; MCV, Mean corpuscular volume; Na, Sodium; PLT, Platelet; RBC, Red blood count; WBC, White blood count; ^a^ The training set included the data from Taipei Medical University and Wan-Fang hospitals; ^b^ The testing set included the data from Shuang Ho hospital.

**Table 2:** Performance of various Prediction Models by Modes.

Modes	Models	AUC Training	AUC Testing	Accuracy	Precision	Recall	F1-score
**Mode 1**	LR	0.70	0.72	0.65	0.88	0.64	0.75
	LDA	0.78	0.78	0.71	0.90	0.70	0.80
	LGBM	0.98	0.81	0.73	0.92	0.72	0.81
	GBM	0.96	0.83	0.75	0.91	0.76	0.84
	XGBoost	0.99	0.80	0.75	0.90	0.77	0.84
	RF	0.90	0.82	0.72	0.92	0.70	0.80
	AdaBoost	0.94	0.81	0.73	0.91	0.72	0.81
	SVC	0.78	0.78	0.71	0.89	0.72	0.79
	ANN *	**0.89**	**0.88**	**0.82**	**0.90**	**0.75**	**0.64**
**Mode 2**	LR	0.74	0.75	0.60	0.93	0.53	0.67
	LDA	0.81	0.79	0.71	0.90	0.70	0.80
	LGBM	0.99	0.83	0.78	0.91	0.79	0.86
	GBM	0.96	0.84	0.78	0.91	0.80	0.87
	XGBoost	1.00	0.81	0.78	0.90	0.81	0.86
	RF	0.92	0.83	0.69	0.94	0.64	0.76
	AdaBoost	0.95	0.80	0.74	0.90	0.76	0.83
	SVC	0.81	0.79	0.70	0.91	0.68	0.78
	ANN *	**0.89**	**0.89**	**0.80**	**0.91**	**0.75**	**0.64**
**Mode 3**	LR	0.70	0.73	0.65	0.88	0.63	0.74
	LDA	0.80	0.81	0.75	0.91	0.76	0.83
	LGBM	0.98	0.85	0.80	0.92	0.81	0.87
	GBM	0.96	0.85	0.79	0.92	0.79	0.86
	XGBoost	1.00	0.83	0.79	0.91	0.80	0.86
	RF	0.91	0.84	0.72	0.93	0.69	0.80
	AdaBoost	0.95	0.83	0.79	0.91	0.80	0.86
	SVC	0.80	0.81	0.75	0.90	0.75	0.83
	ANN *	**0.89**	**0.89**	**0.83**	**0.89**	**0.81**	**0.64**
**Mode 4**	LR	0.74	0.75	0.61	0.93	0.53	0.67
	LDA	0.83	0.82	0.76	0.90	0.77	0.84
	LGBM	0.99	0.86	0.81	0.92	0.83	0.88
	GBM	0.97	0.85	0.79	0.92	0.81	0.87
	XGBoost	1.00	0.84	0.77	0.92	0.77	0.85
	RF	0.93	0.85	0.75	0.93	0.73	0.82
	AdaBoost	0.96	0.83	0.76	0.92	0.75	0.83
	SVC	0.83	0.81	0.75	0.90	0.76	0.84
	ANN *	**0.89**	**0.89**	**0.82**	**0.91**	**0.75**	**0.65**

**Note**: LR, Logistic Regression; LDA, Linear Discriminant Analysis; LGBM, Light Gradient Boosting Machine; GBM, Gradient Boosting Machine; XGBoost, Extreme Gradient Boosting; RF, Random Forest; SVC, Support Vector Machine; ANN, Artificial Neural Network; *, Best model based on AUC values.

## Data Availability

The authors obtained data from the Taiwan Cancer Registry (TCR) database and the Taipei Medical University Clinical Research Database (TMUCRD).

## Supplementary Materials

The following supporting information can be downloaded at: https://www.mdpi.com/article/10.3390/cancers14225562/s1, Figure S1: Cohort Selection Process; Figure S2: Feature Importance of the GBM Prediction Model of Mode 4; Table S1: Detailed Demographic Characteristics of Cohort Patients; Table S2: Detailed Performance of various Prediction Models by Modes.

## Abbreviations

NSCLC	Non-small cell lung cancer
SCLC	Small cell lung cancer
AI	Artificial intelligence
TCR	Taiwan Cancer Registry
TDR	Taiwan Death Registry
TMUCRD	Taipei Medical University Clinical Research Database
TMUH	Taipei Medical University Hospital
WFH	Wan-Fang Hospital
SHH	Shuang-Ho Hospital
BMI	Body mass index
MI	Myocardial infarction
CHF	Congestive heart failure
PVD	Peripheral vascular disease
CVD	Cardiovascular disease
COPD	Chronic obstructive pulmonary disease
PUD	Peptic ulcer disease
CCI	Charlson Comorbidity Index
BUN	Blood urea nitrogen
PLT	Platelet
LR	Logistic regression
LDA	Linear discriminant analysis
LGBM	Light gradient boosting machine
GBM	Gradient boosting machine
XGBoost	Extreme gradient boosting
RF	Random forest
SVC	Support vector machine
ANN	Artificial neural network
AUC	The area under the receiver operating characteristic curve
PPV	Positive predictive value
NPV	Negative predictive value
SHAP	Shapley additive explanations
