# Supplementary material for: Development and Validation of Novel Deep-Learning Models Using Multiple Data Types for Lung Cancer Survival
Source: Cancers (Basel). 2022 Nov 12;14(22):5562. doi: 10.3390/cancers14225562 (PMC9688689; doi:10.3390/cancers14225562)
Supplement: Supplementary file 1 [file cancers-14-05562-s001.zip › cancers-1955058-supplementary.pdf]

Supplementary Materials

Development and Validation of Machine Learning Algorithms for Lung Cancer Survival based on Real-world Data

FIGURE S1. COHORT SELECTION PROCESS 2

FIGURE S2. FEATURE IMPORTANCE OF THE GBM PREDICTION MODEL OF MODE 4.......... 3

TABLE S1. DETAILED DEMOGRAPHIC CHARACTERISTICS OF COHORT PATIENTS.......... 4

TABLE S2. DETAILED PERFORMANCE OF VARIOUS PREDICTION MODELS BY MODES.......... 11

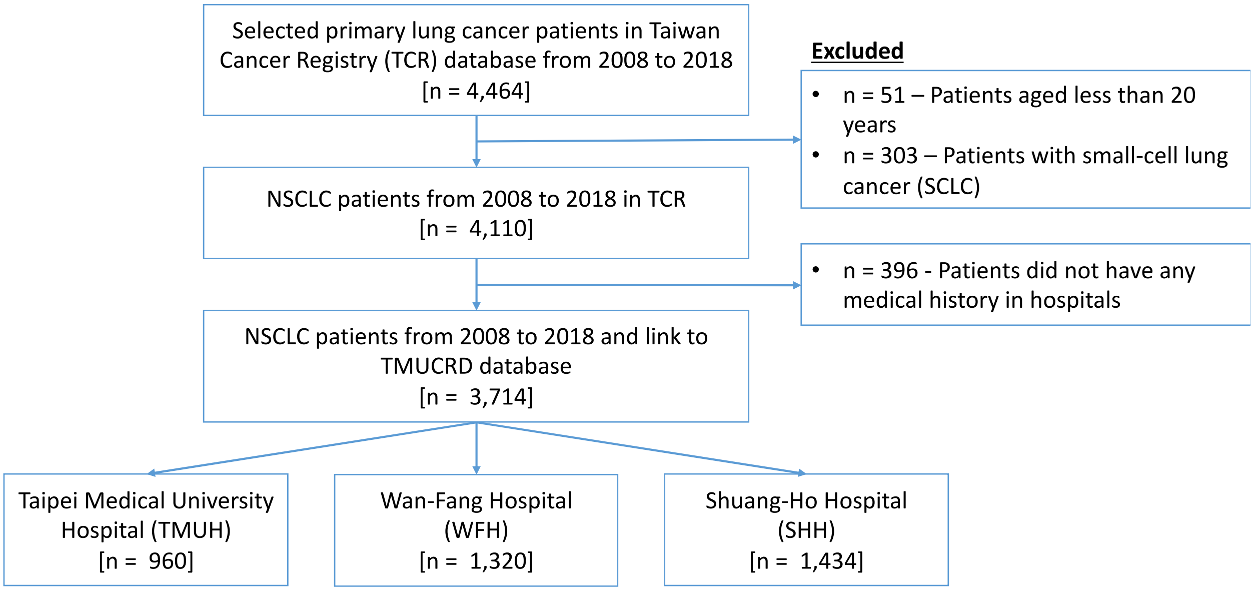

Figure S1. Cohort Selection Process

Note: TCR, Taiwan cancer regisitry; NSCLC, Non-small-cell lung cancer; TMUCRD, Taipei Medical University clinical research database; TMUH, Taipei Medical University hospital; WFH, Wan-Fang hospital; SHH, Shuang-Ho hospital;

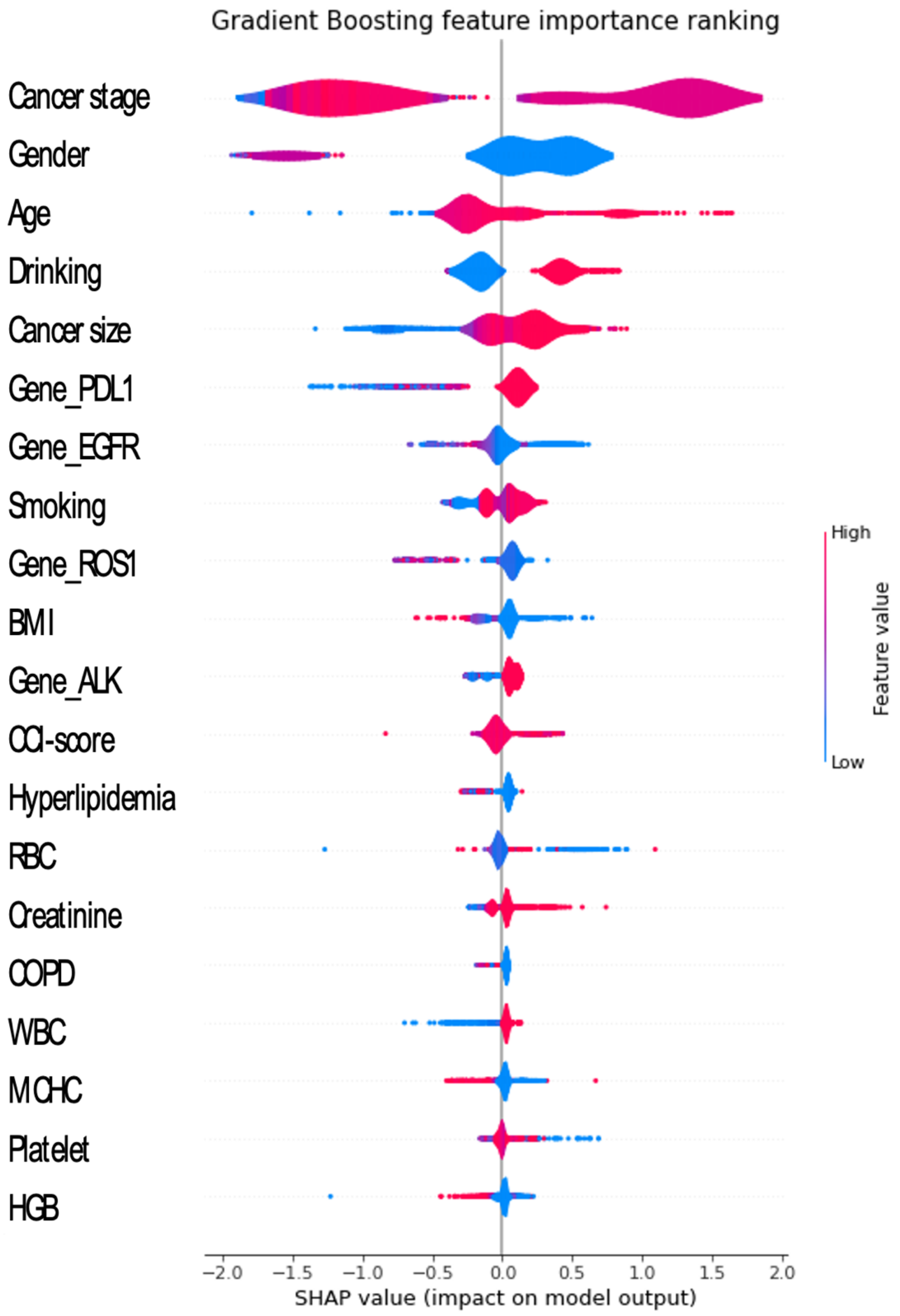

Figure S2. Feature Importance of the GBM Prediction Model of Mode 4

Note: BMI, Body mass index; EGFR, Epidermal growth factor receptor; RBC, Red blood cell; WBC, White blood cell; PD-L1, Programmed death-ligand 1; COPD, Chronic obstructive pulmonary disease; CCI, Charlson comorbidity index; ROS1, Proto-oncogene 1, receptor tyrosine-protein kinase; ALK, Anaplastic lymphoma kinase; MCHC, Mean corpuscular hemoglobin concentration; HGB, Hemoglobin;

Table S1. Detailed Demographic Characteristics of Cohort Patients

	**Overall**<br>**n = 3,714**	**Training cohort[a]**<br>**n = 2,280**	**Testing cohort[b]**<br>**n = 1,434**
**Gender, No. (%)**			
Male	2136 (57.5)	1258 (55.2)	878 (61.2)
Female	1578 (42.5)	1022 (44.8)	556 (38.8)
**Age, Mean (SD), yrs.**			
Mean (SD)	68.0 (13.7)	67.9 (13.8)	68.0 (13.4)
Median [IQR]	68.0 [59 – 79]	68.0 [59 – 79]	68.0 [58 – 78]
**BMI**			
Mean (SD)	23.4 (4.33)	23.4 (3.93)	23.4 (4.81)
Median [IQR]	23.1 [20.8 – 25.5]	23.1 [20.8 – 25.6]	23.0 [20.6 – 25.5]
Unknown	1611 (43.4)	1082 (47.5)	529 (36.9)
**Smoking, No. (%)**			
No	1170 (31.5)	710 (31.1)	460 (32.1)
Yes	993 (26.7)	523 (22.9)	470 (32.8)
Unknown	1551 (41.8)	1047 (45.9)	504 (35.1)
**Drinking, No. (%)**			
No	1750 (47.1)	983 (43.1)	767 (53.5)
Yes	408 (11.0)	247 (10.8)	161 (11.2)
Unknown	1556 (41.9)	1050 (46.1)	506 (35.3)
**Tumor size, cm**			
Mean (SD)	4.23 (2.45)	4.11 (2.39)	4.46 (2.55)
Median [IQR]	3.8 [2.4 – 5.5]	3.6 [2.3 – 5.5]	4.0 [2.5 – 5.7]
Unknown	972 (26.2)	537 (23.6)	435 (30.3)
**Cancer stage, No. (%)**			
stage = 0	11 (0.3)	10 (0.4)	1 (0.1)
stage = 1	533 (14.4)	348 (15.3)	185 (12.9)
stage = 2	139 (3.7)	88 (3.9)	51 (3.6)
stage = 3	527 (14.2)	330 (14.5)	197 (13.7)
stage = 4	2034 (54.8)	1207 (52.9)	827 (57.7)
Unknown	470 (12.7)	297 (13.0)	173 (12.1)

Table S1. Detailed Demographic Characteristics of Cohort Patients (Continued)

	**Overall**<br>**n = 3,714**	**Training cohort[a]**<br>**n = 2,280**	**Testing cohort[b]**<br>**n = 1,434**
**Genomic Test**			
**ALK, N (%)**			
Negative	681 (18.3)	457 (20.0)	224 (15.6)
Positive	39 (1.1)	21 (0.9)	18 (1.3)
Unknown	2994 (80.6)	1802 (79.0)	1192 (83.1)
**EGFR, N (%)**			
Negative	842 (22.7)	473 (20.7)	369 (25.7)
Positive	787 (21.2)	467 (20.5)	320 (22.3)
Unknown	2085 (56.1)	1340 (58.8)	745 (52.0)
**KRAS, N (%)**			
Negative	45 (1.2)	32 (1.4)	13 (0.9)
Positive	5 (0.1)	2 (0.1)	3 (0.2)
Unknown	3664 (98.7)	2246 (98.5)	1418 (98.9)
**PDL1, N (%)**			
Negative	269 (7.2)	149 (6.5)	120 (8.4)
Positive	66 (1.8)	42 (1.8)	24 (1.7)
Unknown	3379 (91.0)	2089 (91.6)	1290 (90.0)
**ROS1, N (%)**			
Negative	288 (7.8)	287 (12.6)	1 (0.1)
Positive	29 (0.8)	27 (1.2)	2 (0.1)
Unknown	3397 (91.4)	1966 (86.2)	1431 (99.8)

Table S1. Detailed Demographic Characteristics of Cohort Patients (Continued)

	**Overall**<br>**n = 3,714**	**Training cohort[a]**<br>**n = 2,280**	**Testing cohort[b]**<br>**n = 1,434**
**Comorbidity, N (%)**			
CVD problems	432 (11.6)	296 (13.0)	136 (9.5)
Dementia	124 (3.3)	71 (3.1)	53 (3.7)
COPD	599 (16.1)	391 (17.1)	208 (14.5)
Rheumatic disease	28 (0.75)	16 (0.7)	12 (0.8)
PUD	365 (9.8)	246 (10.8)	119 (8.3)
Renal disease	128 (3.4)	92 (4.0)	31 (2.2)
Liver disease	211 (5.7)	147 (6.4)	64 (4.5)
DM	372 (10.0)	248 (10.9)	124 (8.6)
Anemia	107 (2.9)	76 (3.3)	31 (2.2)
Depression	245 (6.6)	175 (7.7)	70 (4.9)
Hyperlipidemia	516 (13.9)	385 (16.9)	131 (9.1)
Hypertension	736 (19.8)	503 (22.1)	233 (16.2)
Parkinson's disease	50 (1.3)	29 (1.3)	21 (1.5)
**Charlson Comorbidity Index (CCI)**			
Mean (SD)	3.08 (2.07)	3.13 (2.19)	2.97 (1.86)
Median [IQR]	3.0 [2.0 – 4.0]	3.0 [2.0 – 4.0]	3.0 [2.0 – 4.0]
**Follow-up, yrs.**			
Mean (SD)	2.25 (2.47)	2.44 (2.61)	1.96 (2.19)
Median [IQR]	1.41 [0.46 – 3.04]	1.51 [0.53 – 3.36]	1.24 [0.38 – 2.64]

Table S1. Detailed Demographic Characteristics of Cohort Patients (Continued)

	Overall n = 3,714	Training cohort[a] n = 2,280	Testing cohort[b] n = 1,434
**Laboratory test result**			
**Basophil**			
Mean (SD)	0.51 (0.40)	0.53 (0.42)	0.484 (0.39)
Median [IQR]	0.40 [0.2 – 0.7]	0.50 [0.3 – 0.7]	0.40 [0.2 – 0.7]
Missing	2450 (66.0)	1648 (72.3)	802 (55.9)
**BUN**			
Mean (SD)	19.4 (14.9)	18.8 (13.1)	20.5 (17.6)
Median [IQR]	16.0 [12 – 21]	15.8 [12 – 21]	16.0 [11.4 – 22]
Missing	2256 (60.7)	1350 (59.2)	906 (63.2)
**Creatinine**			
Mean (SD)	1.05 (0.98)	1.02 (0.90)	1.10 (1.07)
Median [IQR]	0.86 [0.7 – 1.08]	0.85 [0.7 – 1.07]	0.86 [0.7 – 1.09]
Missing	1442 (38.8)	957 (42.0)	485 (33.8)
**Eosinophil**			
Mean (SD)	1.89 (2.31)	2.03 (2.59)	1.76 (1.97)
Median [IQR]	1.20 [0.4 – 2.5]	1.30 [0.5 – 2.7]	1.10 [0.4 – 2.4]
Missing	2450 (66.0)	1648 (72.3)	802 (55.9)
**HCT**			
Mean (SD)	38.3 (5.69)	38.5 (5.61)	37.9 (5.80)
Median [IQR]	38.7 [35.0 – 42.2]	39.0 [35.2 – 42.5]	38.1 [34.4 – 41.8]
Missing	1914 (51.5)	1188 (52.1)	726 (50.6)
**HGB**			
Mean (SD)	12.9 (1.97)	13.0 (1.91)	12.7 (2.05)
Median [IQR]	13.0 [11.6 – 14.3]	13.1 [11.7 – 14.4]	12.9 [11.4 – 14.2]
Missing	1854 (49.9)	1135 (49.8)	719 (50.1)
**K**			
Mean (SD)	3.99 (0.56)	4.02 (0.53)	3.95 (0.60)
Median [IQR]	4.00 [3.6 – 4.3]	4.00 [3.7 – 4.3]	3.90 [3.6 – 4.3]
Missing	2140 (57.6)	1325 (58.1)	815 (56.8)
**Lymphocyte**			
Mean (SD)	18.7 (9.98)	19.6 (9.55)	17.8 (10.3)
Median [IQR]	17.5 [11.1 – 25.1]	18.8 [12.7 – 25.6]	16.4 [10.0 – 24.4]
Missing	2450 (66.0)	1648 (72.3)	802 (55.9)
**MCH**			
Mean (SD)	29.9 (3.02)	29.9 (3.03)	29.8 (3.00)
Median [IQR]	30.3 [28.9 – 31.6]	30.4 [29 – 31.7]	30.2 [28.7 – 31.6]
Missing	1996 (53.7)	1259 (55.2)	737 (51.4)
**MCHC**			
Mean (SD)	33.6 (0.95)	33.7 (0.96)	33.6 (0.94)
Median [IQR]	33.7 [33.1 – 34.2]	33.7 [33.2 – 34.3]	33.6 [33.1 – 34.2]
Missing	1996 (53.7)	1259 (55.2)	737 (51.4)

**MCV**			
Mean (SD)	88.6 (7.61)	88.5 (7.64)	88.7 (7.57)
Median [IQR]	89.5 [86 – 93.1]	89.4 [86 – 93.3]	89.7 [86.2 - 93]
Missing	1947 (52.4)	1219 (53.5)	728 (50.8)
**Monocyte**			
Mean (SD)	7.45 (2.90)	7.42 (2.93)	7.48 (2.87)
Median [IQR]	7.40 [5.6 – 9.2]	7.40 [5.5 – 9.2]	7.35 [5.6 – 9.2]
Missing	2450 (66.0)	1648 (72.3)	802 (55.9)
**Na**			
Mean (SD)	137 (4.46)	137 (4.39)	137 (4.53)
Median [IQR]	138 [135 - 140]	138 [135 - 140]	137 [134 - 139]
Missing	2200 (59.2)	1373 (60.2)	827 (57.7)
**Neutrophil**			
Mean (SD)	71.3 (11.9)	70.2 (11.4)	72.3 (12.2)
Median [IQR]	71.9 [63.6 – 80]	70.6 [63 – 77.9]	73.1 [64 – 82]
Missing	2450 (66.0)	1648 (72.3)	802 (55.9)
**PLT**			
Mean (SD)	263 (109)	258 (100)	269 (121)
Median [IQR]	245 [196 – 308.8]	243 [197 – 300]	250 [194 – 320]
Missing	1900 (51.2)	1167 (51.2)	733 (51.1)
**RBC**			
Mean (SD)	4.35 (0.68)	4.38 (0.67)	4.29 (0.69)
Median [IQR]	4.37 [3.93 – 4.78]	4.41 [3.97 – 4.81]	4.32 [3.87 – 4.75]
Missing	1934 (52.1)	1206 (52.9)	728 (50.8)
**WBC**			
Mean (SD)	9.72 (5.38)	9.16 (4.16)	10.6 (6.80)
Median [IQR]	8.55 [6.7 – 11.5]	8.19 [6.47 – 10.9]	9.00 [6.9 – 12.4]
Missing	1861 (50.1)	1142 (50.1)	719 (50.1)

Table S1. Detailed Demographic Characteristics of Cohort Patients (Continued)

	Overall n = 3,714	Training cohort[a] n = 2,280	Testing cohort[b] n = 1,434
**Medication, N (%)**			
**Alimentary tract and metabolism**	591 (15.9)	394 (17.3)	197 (14.7)
A02AA - Magnesium compounds	61 (1.6)	37 (1.6)	24 (1.7)
A02AX - Antacids, other combinations	80 (2.2)	61 (2.7)	19 (1.3)
A02BA - H2-receptor antagonists	115 (3.1)	69 (3.0)	46 (3.2)
A02BC - Proton pump inhibitors	107 (2.9)	66 (2.9)	41 (2.9)
A03AX - Other drugs for functional gastrointestinal disorders	79 (2.1)	57 (2.5)	22 (1.5)
A03FA - Propulsives	114 (3.1)	65 (2.9)	49 (3.4)
A06AB - Contact laxatives	215 (5.8)	163 (7.1)	52 (3.6)
A06AD - Osmotically acting laxatives	52 (1.4)	42 (1.8)	10 (0.7)
A10BA - Biguanides	165 (4.4)	118 (5.2)	47 (3.3)
A10BB - Sulfonylureas	114 (3.1)	78 (3.4)	36 (2.5)
A10BH - Dipeptidyl peptidase 4 (DPP-4) inhibitors	92 (2.5)	66 (2.9)	26 (1.8)
**Blood and blood-forming organs**	446 (12.0)	293 (12.9)	153 (11.3)
B01AC - Platelet aggregation inhibitors	420 (11.3)	277 (12.1)	143 (10.0)
B03BA - Vitamin B12 (cyanocobalamin and analogues)	56 (1.5)	33 (1.4)	23 (1.6)
**Cardiovascular system**	675 (18.2)	448 (19.6)	227 (16.9)
C01DA - Organic nitrates	103 (2.8)	72 (3.2)	31 (2.2)
C01DX - Other vasodilators used in cardiac diseases	103 (2.8)	65 (2.9)	38 (2.7)
C02CA - Alpha-adrenoreceptor antagonists	58 (1.6)	35 (1.5)	23 (1.6)
C03AA - Thiazides, plain	63 (1.7)	42 (1.8)	21 (1.5)
C03CA - Sulfonamides, plain	99 (2.7)	61 (2.7)	38 (2.7)
C04AD - Purine derivatives	73 (2.0)	45 (2.0)	28 (2.0)
C07AB - Beta blocking agents, selective	172 (4.6)	111 (4.9)	61 (4.3)
C07AG - Alpha and beta blocking agents	75 (2.0)	59 (2.6)	16 (1.1)
C08CA - Dihydropyridine derivatives	271 (7.3)	174 (7.6)	97 (6.8)
C08DB - Benzothiazepine derivatives	83 (2.2)	64 (2.8)	19 (1.3)
C09CA - Angiotensin II receptor blockers (ARBs), plain	288 (7.8)	191 (8.4)	97 (6.8)
C10AA - HMG CoA reductase inhibitors	294 (7.9)	211 (9.3)	83 (5.8)
**Genitourinary system and hormones**	132 (3.6)	74 (3.2)	58 (4.3)
G04CA - Alpha-adrenoreceptor antagonists	132 (3.6)	74 (3.2)	58 (4.0)
**Musculo-skeletal system**	252 (6.8)	141 (6.2)	111 (8.3)
M01AH - Coxibs	108 (2.9)	53 (2.3)	55 (3.8)
M02AA - Antiinflammatory preparations, non-steroids for topical use	92 (2.5)	43 (1.9)	49 (3.4)
M03BB - Oxazol, thiazine, and triazine derivatives	52 (1.4)	27 (1.2)	25 (1.7)
M04AB - Preparations increasing uric acid excretion	63 (1.7)	45 (2.0)	18 (1.3)

**Nervous system**	391 (10.5)	254 (11.1)	137 (10.2)
N03AE - Benzodiazepine derivatives (Antiepileptics)	61 (1.6)	35 (1.5)	26 (1.8)
N05BA - Benzodiazepine derivatives (Anxiolytics)	167 (4.5)	118 (5.2)	49 (3.4)
N05CD - Benzodiazepine derivatives (Hypnotics and sedatives)	81 (2.2)	50 (2.2)	31 (2.2)
N05CF - Benzodiazepine related drugs (Hypnotics and sedatives)	69 (1.9)	40 (1.8)	29 (2.0)
N06BX - Other psychostimulants and nootropics	65 (1.8)	39 (1.7)	26 (1.8)
N07CA - Antivertigo preparations	77 (2.1)	51 (2.2)	26 (1.8)
**Respiratory system**	319 (8.6)	226 (9.9)	93 (6.9)
R03CC - Selective beta-2-adrenoreceptor agonists	50 (1.3)	37 (1.6)	13 (0.9)
R03DA - Xanthines	156 (4.2)	102 (4.5)	54 (3.8)
R05CB - Mucolytics	185 (5.0)	136 (6.0)	49 (3.4)
R05DA - Opium alkaloids and derivatives	63 (1.7)	36 (1.6)	27 (1.9)
R05FA - Opium derivatives and expectorants	78 (2.1)	50 (2.2)	28 (2.0)
R06AX - Other antihistamines for systemic use	66 (1.8)	49 (2.1)	17 (1.2)

Note: SD, Standard deviation; yrs., Years; IQR, Interquartile Range; BMI, Body mass index; COPD, Chronic obstructive pulmonary disease; PUD, Peptic ulcer disease; CVD, Cardiovascular; DM, Diabetes; BUN, Blood urea nitrogen; HCT, Hematocrit; HGB, Hemoglobin; K, Potassium; MCH, Mean corpuscular hemoglobin; MCHC, Mean corpuscular hemoglobin concentration; MCV, Mean corpuscular volume; Na, Sodium; PLT, Platelet; RBC, Red blood count; WBC, White blood count;

[a]The training set included the data from Taipei Medical University and Wan-Fang hospitals;

[b]The testing set included the data from Shuang Ho hospital;

Table S2. Detailed Performance of various Prediction Models by Modes

Modes	Models	Training_AUC	Testing_AUC	Accuracy	Sensitivity	Specificity	PPV	NPV	F1-score
**Mode 1**	LR	0.70	0.72	0.65	0.64	0.69	0.88	0.36	0.75
	LDA	0.78	0.78	0.71	0.70	0.72	0.90	0.42	0.80
	LGBM	0.98	0.81	0.73	0.72	0.77	0.92	0.45	0.81
	GBM	0.96	0.83	0.75	0.76	0.74	0.91	0.47	0.84
	XGBoost	1.00	0.80	0.75	0.77	0.70	0.90	0.47	0.84
	RF	0.90	0.82	0.72	0.70	0.79	0.92	0.43	0.80
	Ada Boost	0.94	0.81	0.73	0.72	0.75	0.91	0.44	0.81
	SVC	0.78	0.78	0.71	0.72	0.70	0.89	0.42	0.81
	**ANN***	**0.89**	**0.88**	**0.82**	**0.75**	**0.71**	**0.90**	**0.45**	**0.64**
**Mode 2**	LR	0.74	0.75	0.60	0.53	0.85	0.93	0.35	0.67
	LDA	0.81	0.79	0.71	0.70	0.74	0.90	0.42	0.80
	LGBM	0.99	0.83	0.78	0.79	0.73	0.91	0.51	0.86
	GBM	0.96	0.84	0.78	0.80	0.71	0.91	0.51	0.87
	XGBoost	1.00	0.81	0.78	0.81	0.68	0.90	0.51	0.86
	RF	0.92	0.83	0.69	0.64	0.86	0.94	0.41	0.76
	Ada Boost	0.95	0.80	0.74	0.76	0.70	0.90	0.46	0.83
	SVC	0.81	0.79	0.70	0.68	0.77	0.91	0.41	0.78
	**ANN***	**0.89**	**0.89**	**0.80**	**0.75**	**0.74**	**0.91**	**0.46**	**0.64**
**Mode 3**	LR	0.70	0.73	0.65	0.63	0.70	0.88	0.36	0.74
	LDA	0.80	0.81	0.75	0.76	0.73	0.91	0.47	0.83
	LGBM	0.98	0.85	0.80	0.81	0.75	0.92	0.54	0.87
	GBM	0.96	0.85	0.79	0.79	0.77	0.92	0.52	0.86
	XGBoost	1.00	0.83	0.79	0.80	0.74	0.91	0.52	0.86
	RF	0.91	0.84	0.72	0.69	0.83	0.93	0.44	0.80
	Ada Boost	0.95	0.83	0.79	0.80	0.73	0.91	0.52	0.86
	SVC	0.80	0.81	0.75	0.75	0.72	0.90	0.46	0.83
	**ANN***	**0.89**	**0.89**	**0.83**	**0.81**	**0.67**	**0.89**	**0.50**	**0.64**

**Mode 4**	LR	0.74	0.75	0.61	0.53	0.85	0.93	0.35	0.67
	LDA	0.83	0.82	0.76	0.77	0.72	0.90	0.48	0.84
	LGBM	0.99	0.86	0.81	0.83	0.74	0.92	0.55	0.88
	GBM	0.97	0.85	0.79	0.81	0.75	0.92	0.53	0.87
	XGBoost	1.00	0.84	0.77	0.77	0.78	0.92	0.49	0.85
	RF	0.93	0.85	0.75	0.73	0.81	0.93	0.46	0.82
	Ada Boost	0.96	0.83	0.76	0.75	0.77	0.92	0.48	0.83
	SVC	0.83	0.81	0.75	0.76	0.72	0.90	0.46	0.84
	**ANN***	**0.89**	**0.89**	**0.82**	**0.75**	**0.73**	**0.91**	**0.46**	**0.65**

Note: LR, Logistic Regression; LDA, Linear Discriminant Analysis; LGBM, Light Gradient Boosting Machine; GBM, Gradient Boosting Machine; XGBoost, Extreme Gradient Boosting; RF, Random Forest; SVC, Support Vector Machine; ANN, Artificial Neural Network; *, Best model based on AUC values;
